# Intravenous leiomyomatosis of the uterus with extension to the right heart

**DOI:** 10.1186/1476-7120-9-25

**Published:** 2011-09-24

**Authors:** Yu-Feng Lou, Xin-Ping Shi, Ze-Zhou Song

**Affiliations:** 1 Department of Laboratory, The First Affiliated Hospital, College of Medicine, Zhejiang University, Hangzhou, China; 2 Department of Laboratory, Tong-De Hospital of Zhejiang Province, Hangzhou, China; 3 Department of Ultrasound, The First Affiliated Hospital, College of Medicine, Zhejiang University, Hangzhou, China

## Abstract

A 42-year-old woman admitted with debilitation and engorgement both lower extremities. Transthoracic two-dimensional echocardiography, abdominal ultrasound and computerized tomography revealed a lobulated pelvic mass, a mass within right internal iliac vein, both common iliac vein, as well as the inferior vena cava, extending into the right atrium. In addition, echocardiography and abdominal ultrasound showed the tumor of right atrium and inferior vena cave has no stalk and has well-demarcated borders with the wall of right atrium and inferior vena cave. Hence, the presumptive diagnosis of IVL was made by echocardiography and abdominal ultrasound and the presumptive diagnosis of sarcoma with invasion in right internal iliac vein, both common iliac vein, the inferior vena cava, as well as the right atrium was made by multi-detector-row computerized tomography. The patient underwent a one-stage combined multidisciplinary thoraco-abdominal operation under general anaesthetic. Subsequently the pathologic report confirmed IVL.

## Introduction

Benign smooth muscle neoplasms or leiomyomas are extremely common uterine tumors. On rare occasions, the neoplasms exhibit unusual growth patterns [1]. One such pattern is intravenous leiomyomatosis (IVL), which is an uncommon neoplasm characterized by intravascular proliferation of a histologically benign-looking smooth muscle cell tumor mass but not invading the tissue. Although IVL is usually confined to the pelvis and histologically benign, IVL exposed to venous blood that flows to the heart and may penetrate the inferior vena cava, reach the right heart chambers, or extend to the right pulmonary artery [2], which is named intracardiac leiomyomatosis and may results in cardiac symptoms and cardiac murmur, fainting and even in some cases, sudden death [3]. Because of rarity, IVL is occasionally misdiagnosed or diagnosed lately, and subsequently improperly treated and correct preoperative diagnosis of IVL depends on a huge index of doubt. Therefore, we present a case of IVL, which was cued by transthoracic echocardiography and abdominal ultrasound and confirmed by histopathological evaluation and review the literature.

## Case Report

The patient was a 42-year-old woman admitted with debilitation and engorgement of both lower extremities. In past, she has undergone hysterectomy one year and six months ago because of hysteromyoma and was never found to have any heart diseases and relevant history. She was never found to have diabetes mellitus and denied relevant history of smoking. On examination, there was no abnormality in physical examination including cardiac auscultation etc. except for mild edema of both lower extremities. The laboratory examinations revealed normal results including tumor markers etc.

**Figure 1:**
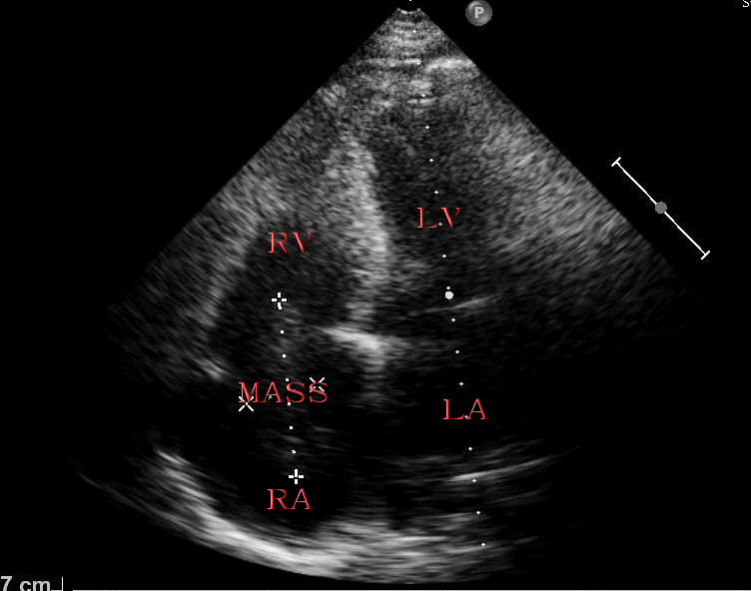
**Apical four chamber view showing right atrium filled with a medium echogenic oval tumor mass by transthoracic two-dimensional echocardiography**. RV: right ventricle, RA: right atrium, LV: left ventricle, LA: left atrium.

**Figure 2:**
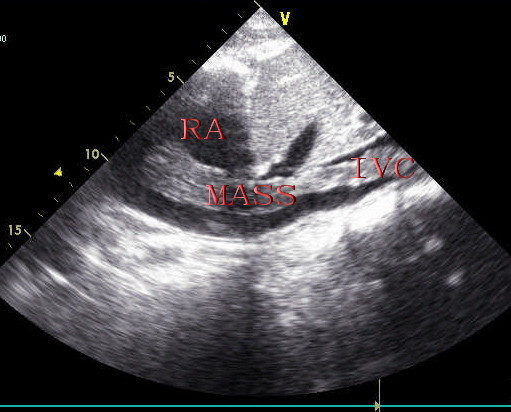
**Under-ensisternum view showing mass originated from inferior vena cave, extended into right atrium and that mass of right atrium and inferior vena cave has no stalk and has well-demarcated borders with the wall of right atrium and inferior vena cave by transthoracic two-dimensional echocardiography**. RA: right atrium, IVC: inferior vena cave.

**Figure 3:**
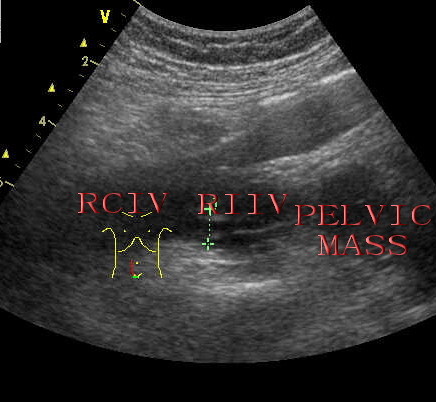
**Pelvic view showing a pelvic mass and continued medium echogenic oval tumor mass within right internal iliac vein, right common iliac vein by abdominal ultrasound**. RIIV: right internal iliac vein, RCIV: right common iliac vein.

Transthoracic two-dimensional echocardiography showed normal parameters of the left and right ventricular wall thickness, size and function. The right atrium was mildly enlarged and filled with a medium echogenic oval tumor mass which is approximately 5.5 cm*2.3 cm (Figure 1). The tumor mass originated from inferior vena cave, extended into right atrium (Figure 2), moved back and forth through the tricuspid orifice into right ventricle. The tumor of right atrium and inferior vena cave has no stalk and has well-demarcated borders with the wall of right atrium and inferior vena cave (Figure 2), which caused the tumor wandering within right atrium and inferior vena cave. The moderate tricuspid regurgitation was detected. In addition, abdominal ultrasound revealed a pelvic mass and continued medium echogenic oval tumor mass within right internal iliac vein, both common iliac vein, as well as the inferior vena cava (Figure 3). Hence, the presumptive diagnosis of IVL was made by echocardiography and abdominal ultrasound.

**Figure 4:**
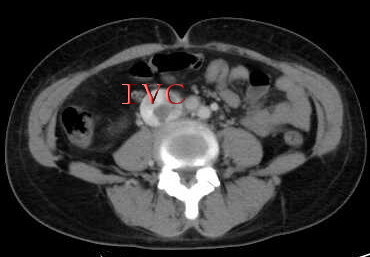
**Abdominal computerized tomography revealed a low attenuation-filling continued defect within the inferior vena cava**. IVC: inferior vena cava.

**Figure 5:**
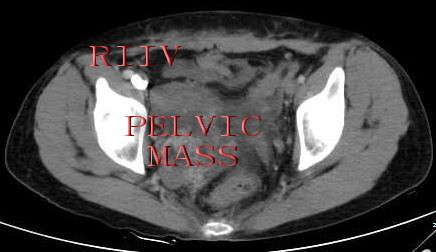
**Pelvic computerized tomography revealed a lobulated pelvic mass, a low attenuation-filling continued defect within right internal iliac vein**. RIIV: right internal iliac vein.

The multi-detector-row spiral plain scan and contrast-enhanced computerized tomography of thoracic cavity, abdominal cavity and pelvic cavity, which was clearly visualized in the present case, revealed a lobulated pelvic mass, a low attenuation-filling continued defect was noted from an enlarged right internal iliac vein, both common iliac vein, as well as within the inferior vena cava, extending into the right atrium (Figure 4,5) as the features of echocardiography and abdominal ultrasound. Therefore, the presumptive diagnosis of sarcoma with invasion in right internal iliac vein, both common iliac vein, the inferior vena cava, as well as the right atrium was made by multi-detector-row computerized tomography.

The patient underwent a one-stage combined multidisciplinary including department of gynaecology, cardiac surgery and vascular surgery thoraco-abdominal operation under general anaesthetic. The pelvic mass was excised by gynaecological team. The tumor within vascular and heart was upwardly dislodged by opening the right atrium. The length of tumor within vascular and heart was 25 cm. Consistent with the features of echocardiography and abdominal ultrasound, the surgical views revealed that the tumor within vascular and heart has no stalk and has well-demarcated borders with the wall of vascular and heart. Subsequently the pathologic report confirmed IVL.

## Discussion

IVL is histologically confirmed benign smooth muscle tumor within vascular spaces from intrauterine venules to the systemic veins including iliac vein, the inferior vena cava, even extending into the right heart chamber and pulmonary artery and its extrauterine involvement occurs in approximately 30% of cases and intracardiac extension accounts for about 10% [4,5]. Birch-Hirschfeld [6] first presented case of IVL in 1896 and Durck [7] first presented case of intracardiac extension of IVL in 1907. Although the pathogenesis of IVL remain unclear, there are two main theories regarding the etiology of IVL. The first theory is that IVL is leiomyoma originated from the venous wall [8] and the other is that IVL is the uterine leiomyoma which invaded into the uterine vein [9]. In the present case there was evidence of uterine leiomyoma invading into the vessels provided by the feature of echocardiography, abdominal ultrasound and computerized tomography. As detected by echocardiography and abdominal and viewed by surgery, the intravascular and intra-atrium tumor mass were not attached to the endothelial surface and endocardium but were freely movable within the vascular and heart spaces, which was easily removed. These features suggest that IVL may be the uterine leiomyoma which invaded into the uterine vein in the present cases. Up to date, the mechanisms of diffusion to the heart in IVL patients remain unclear. However, incomplete surgery hysterectomy has been presumed to promote the proliferation of IVL. In addition, Yaguchi C et al [10] reported that hyaluronan, which is an important constituent of the extracellular matrix and is known to regulate cellular events, was expressed more prominently in IVL than in uterine leiomyomas by immunohistochemical studies and propose that IVL has viscoelastic properties and contains a large amount of hyaluronan, which may promote invasion during pathogenesis.

IVL is usually occurring in women of ages 23 to 80 (median age, 44 years) [11]. Most patients, including the present case, have a history of hysterectomy. It is presumed that IVL had simply not been diagnosed in the period of time immediately following the hysterectomy. A tumor left inside the small pelvic veins at the time of hysterectomy [12], which could not be detected by all kinds of imaging methods including ultrasound, computerized tomography and MRI etc., could have extended towards the right heart, leading to obstruction. Kuenen BC et al [13] reported that there was 4-year median interval from performing hysterectomy to the diagnosis of IVL with extension to heart and differences in cytological features only related to the time period of the interval from performing hysterectomy to the diagnosis of IVL with extension to heart [14]. Therefore, the present case could have more athletic cytological features.

The clinical characteristics of IVL are usually similar to typical uterine leiomyomas and relates to the obstructive effect of the tumor on the tricuspid orifice and disturbance of venous return, which could include dyspnea on exertion, syncopal episodes, pulmonary embolism, and sudden death [15-17]. The current patient presented with debilitation and engorgement of both lower extremities. The presence of debilitation and engorgement of both lower extremities motivated echocardiographic and abdominal ultrasonographic examination revealing the presence of a right heart chamber and the inferior vena cava tumor mass. To reveal the features of Chinese IVL patients, The clinical features, treatment of the IVL patients with extension to heart in China like the present case from 2001 to 2011 (in that studies all the patients have detailed clinical parameters) are summarized by exploring the Pubmed in Table 1 to display the features of Chinese IVL patients [18-20]. As same as the present case, the Chinese IVL patients usually have the history of hysterectomy.

**Table 1:** The clinical features and treatment of Chinese IVL patients with extension to heart

Case	Age	Presentation	History of hysterectomy	Treatment	Follow-up without recurrence
1	52	Shortness of breath and palpitation^18^	No data	Two-stage operation	Two-year
2	49	Palpitation^18^	No data	Two-stage operation	Six-month
3	39	Chest tightness and dyspnea^19^	Yes	Two-stage operation	No data
4	40	Dyspnea on exertion and pedal edema^20^	Yes	Two-stage operation	Six-month

Due to its rarity, the diagnosis of IVL is often overlooked. Echocardiography, abdominal ultrasound, computerized tomography and MRI etc. are available for the detection and diagnosis of IVL. When an abdominal pelvic intravenous mass is demonstrated, different diagnosis include abdominal tumors with metastasis to the systemic vein, such as hepatocellular carcinoma, renal cell carcinoma, adrenal tumor and leiomyosarcoma of the uterus etc., bland thrombus, or leiomyosarcoma of the inferior vena cava [21,22]. The features and attachment site of the tumor detected by echocardiography, abdominal ultrasound, computerized tomography and MRI may offer important information regarding the nature of the tumor. The presence of a long, serpentine, polypoidal and elongated mobile mass extending from the inferior vena cava or the veins of the lower body into the right atrium should raise the suspicion of IVL [23,24]. Of course, direct connection between the intravenous mass and the uterus or adnexa, and absence of renal, hepatic or adrenal masses, favor the diagnosis of IVL. In the present case, ultrasound and computerized tomography revealed a long, serpentine, polypoidal and elongated mobile mass extending from right internal iliac vein continued to the inferior vena cava extending into the right atrium and the mass related to pelvic mass at absence of renal, hepatic or adrenal masses. So the ultrasonographer raised the presumptive diagnosis of IVL. To our knowledge, this is the first case which was preoperatively confirmed. Of course, the thrombus within right heart chamber atrium and inferior vena cava can also be mobile or free-floating. Contrast-enhanced computerized tomography or MRI can aid in the different diagnosis of IVL and thrombus.

To date, the complete surgical excision is the first and best treatment of choice for IVL, although antiestrogen therapy had also been proposed [25]. IVL usually adhere to but do not invade the vessel wall and therefore, can usually be removed by downward traction from the ovarian vein, iliac vein or inferior vena cava. However, a combined multidisciplinary thoraco-abdominal operation with removal cardiopulmonary bypass and the removal of tumour in the inferior vena cava and right heart chamber is indicated if IVL is extending to the right heart chamber. In the present case, the intra-cardiac and intra-vascular mass was free-floating without involvement of the cardiac structure and vein wall tissue. Therefore, surgery was performed by a one-stage operation, involving upward dislodgment of the intra-cardiac and intra-vascular tumor by opening the right atrium and resection of the pelvic tumor. Of course, the two-stage operation seems usually to be performed in Chinese patients with IVL and extension to heart and there are no recurrences in short period.

## Competing interests

The authors declare that they have no competing interests.

## Consent

Written informed consent was obtained from the patient for publication of this case report and accompanying images. A copy of the written consent is available for review by the Editor-in-Chief of this journal.

## Authors' contributions

YFL collected the related information, carried out laboratory examination and drafted the manuscript. ZZS carried out Echocardiographic examination and revised the manuscript. All authors read and approved the final manuscript.
